# New occurrence records on the rodent species inhabiting Vietnam, based on Joint Russian-Vietnamese Tropical Research and Test Center genetic samples collection

**DOI:** 10.3897/BDJ.10.e96062

**Published:** 2022-11-23

**Authors:** Alexander E Balakirev

**Affiliations:** 1 A.N.Severtsov's Institute of Ecology and Evolution of Russian Academy of Sciences, Moscow, Russia A.N.Severtsov's Institute of Ecology and Evolution of Russian Academy of Sciences Moscow Russia; 2 Joint Russian-Vietnamese Tropical Research and Test Center, Hanoi, Vietnam Joint Russian-Vietnamese Tropical Research and Test Center Hanoi Vietnam

**Keywords:** mammals, rodents, Muridae, Sciuridae, Spalacidae, Scandentia, Tupaidae, Tupaia, Southeast Asia, biodiversity, species, distribution, occurrence records, Vietnam, genetic samples collection.

## Abstract

**Background:**

Open access to occurrence records in a standardised format has strong potential applications for many kinds of ecological research and bioresources management, including the assessment of invasion risks, formulation of nature protection, biomedical and management plans in the context of global climate and land-use changes both in the short and long perspective. The accumulation and aggregation of data on the occurrence records of small mammals are relevant for the study of biogeography and for ecological surveys including construction of the spatial distribution and ecological niche modelling of species ' distributions in the context of global climate change. The author has created a dataset of 2408 rodents and tree shrews occurrence records from Vietnam, collected from November 2007 to May 2022. A number of zoologist colleagues also provided genetic samples. A considerable part of these data has been published previously in a number of papers; however, most of these data have yet to be presented. These records cover a significant part of the range of many rodent species in Southeast Asia and provide new data on their distribution. The data were obtained during a number of different field expeditions, where some animals were caught by the author and some were provided by other researchers, resulting in different accuracy levels of geographic coordinates and altitude estimates may range from 10 to 1000 metres in area and from 1 to 100 metres for elevation. A number of samples were genetically examined to avoid inconsistencies with the taxonomic identification. With the help of colleagues, the author created a set of georeferenced occurrence records, adapted to the controlled vocabulary of Darwin Core format datasets, removed duplicates and standardised the format of records using commonly-used unified data structure. This paper presents the resulting dataset of rodents (mostly of Muridae and Sciuridae) along with other small terrestrial species (Scandentia Tupaidae) occurrence records in the territory of Vietnam and Laos.

**New information:**

Much of the distribution data are currently available as open source GBIF databases and potentially may be combined into a united framework for better data resolution. The dataset presented here combines occurrence records of many species over a significant part of their recent natural range, in Vietnam and Laos. The author presents a validated and comprehensive dataset of rodents' occurrence records, based on genetic samples collection compiled during 15 years working in Vietnam (from 2007 to date). Prior to this project, a considerable part of the information about Vietnamese rodents was not available to a wide range of researchers to use these spatial data for analyses by modern methods, for example, for analysis based on geographic information systems (GIS technologies). This dataset now is available for any researchers who use the data format prepared in accordance with Darwin Core standards.

For different countries of Southeast Asia and beyond, there are a lot of additional occurrence records for a number of species listed here which may be combined, but a considerable part of them is still scattered over a number of separate literary sources, while another is still presented as maps, field notes and huge amount of museum zoological collections records. The final set was created by a combination of species occurrence records and uniform data structure with verification of the samples' geographic coordinates. Most samples were genetically or/and morphologically verified for correct taxonomical identification, because the most part of the samples presented was carefully investigated by the author himself, both for morphology and genetic attribution. Therefore, the dataset expands the available information on the spatial and temporal distribution of a number of small mammals’ species in Southeast Asia. All original notes and geographical localities were carefully checked and any duplicate and erroneous records have been removed from the final dataset.

To the date of publication of these data, the GBIF database https://www.gbif.org contained 1408 rodent occurrence records from Vietnam (Fig. 1) along with 240 Scandentia records (Fig. 2), primarily the data on museum materials, including four large collections, such as the Field Museum of Natural History (Zoology) Mammal Collection (646 samples), Australian National Wildlife Collection provider for OZCAM (537), MVZ Mammal Collection Arctos (109), Museum of Comparative Zoology, Harvard University (69) and six other minor collections comprising single specimens.

Actually, as for the small terrestrial mammals, Vietnam remains one of the least representative regions in Southeast Asia. Here, we present new data containing 2408 occurrence records, including 2237 rodent records, along with 171 Scandentia ones (Fig. 3). Thus, the data significantly expand our knowledge about actual ranges of a number of species, including rare and endangered ones.

## Introduction

Despite the long history of investigations, fauna composition and limits of a number of species and morpha of rodents, composing the bulk of the fauna of small terrestrial mammals in Southeast Asia, remain not completely understood. By the end of the 20^th^ century, based on investigation of the museums' collections and combininginformation on distribution accumulated over the previous period, the first reports on the fauna of the region were compiled and systematised, along with the records for individual countries and territories (Medway 1965, Medway 1969, Van Peenen et al. 1969, Harrison 1974, Marshall 1976, Marshall 1977b, Marshall 1977a, Marshall 1988, Musser 1981, Taylor et al. 1982, Taylor et al. 1983, Sokolov 1982, Sokolov 1986, Sokolov 1992, Musser and Newcomb C. 1983, Heaney 1986, Corbet and Hill 1991, Corbet and Hill 1992, Musser and Carleton 1993, Pavlinov et al. 1995), which made it possible to establish, in general terms, the taxonomy (at least with resolution up to genera), to assess the species composition and formulate the initial ideas about natural ranges of rodents inhabiting Southeast Asia. Based on these papers, several field-guides, popular and reference papers were compiled shortly afterwards (Medway 1977, Medway 1978, Medway 1983, Payne et al. 1985, Lekagul and McNeely 1977, Lekagul and McNeely 1988, Nowak 1991, Nowak 1999, Zhang et al. 1997, Wilson and Reeder 1993, Kuznetsov 2006, Pavlinov 2006, Smith and Xie 2008, Francis 2008); theyhave been used up to recently for taxonomy and practical issues in the field of nature conservation and biodiversity investigation. In spite of the actual advances, fragmentary geography of samples (incomplete coverage), wide polymorphism and morphological similarity of many species of Muridae, primarily amongst the largest genera, such as *Rattus, Niviventer, Chiromyscus, Leopoldamys, Mus* and others, as well as significant morphological variability of a number of groups of Sciuridae, make it difficult to form correct views on the actual richness of small mammals’ fauna in the region and natural ranges of the species. However, the main reason for the scarcity of knowledge in this field, which had developed by the end of the 20^th^ century, is the lack of researchers who are ready to carefully dealwith this very complex and interesting group in a region remote from the centres of western academic science. Despite the actual advances in macrosystematics, the issue of species composition, its limits and ranges within most genera have remained largely vague and unclear for a long time.

By the beginning of 21^th^ century, with genetic methods of analysis, it became possible to clarify many issues about the correspondence of species/morpha previously described under various names, as well as to begin to search and study so-called cryptic species. Genetic techniques, along with classic morphological approaches, made it possible to carry out accurate species diagnostics. Almost immediately, it became obvious that the species richness and fauna composition within the main genera and groups of Muridae in Southeast Asia, such as *Rattus*, *Niviventer*, *Leopoldamys*, *Maxomys*, *Bandicota* and *Mus*, is considerably underestimated, the same being true about most of the smaller and more exotic groups of mice and rats, such as *Typhlomys*, *Chiropodomys*, *Chiromyscus*, *Dacnomys*, *Saxatilomys* and *Tonkinomys* and the same is true for most of the Sciuridae genera, including the largest squirrel genera like *Callosciurus*, *Tamiops* and *Dremomys*.

The surveys of the author in Vietnam in the period 2007-2022 years were mainly devoted to clarifying the taxonomy and systematics of the main groups of small terrestrial rodents and resulted in a number of generic revisions and descriptions of new taxa (Balakirev and Rozhnov 2010, Balakirev and Rozhnov 2012, Balakirev and Rozhnov 2019; Balakirev et al. 2011a, Balakirev et al. 2011b, Balakirev et al. 2012, Balakirev et al. 2013, Balakirev et al. 2013b, Balakirev et al. 2014, Balakirev and Rozhnov 2019, Balakirev et al. 2021, Balakirev et al. 2022, Balakirev et al. 2017, Abramov et al. 2014, Abramov et al. 2017a, Abramov et al. 2017b) and, simultaneously, the author composed the collection of genetic (and corresponding morphological) samples held in A.N. Seversov’s Institute of Ecology and Evolution of Russian Academy of Sciences (Moscow, Russia) and its international department Joint Russian-Vietnamese Tropical Research and Test Center (Hanoi, Vietnam). On this basis, it was possible to complete the occurrence database, which is presented in this paper.

## General description

### Purpose

The presented data are the most important basis for the study of natural biodiversity, including its dynamics and the investigation of the processes of formation of the mammalian fauna both in evolutionary and historical time-scales. These records are also important for the development of ecological niche models to study the correlation between climate and land-use parameter changes and the participation of recent species in complex fauna through space and time. The publication of occurrence records, based on aggregated data in a standardised format, may also provide valid information and contribute to research of the biological invasion processes.

## Project description

### Title

Small terrestrial mammals of eastern Indochina (Vietnam and neighbouring countries).

### Personnel

Alexander E. Balakirev PhD.

### Study area description

**Figure 4.**
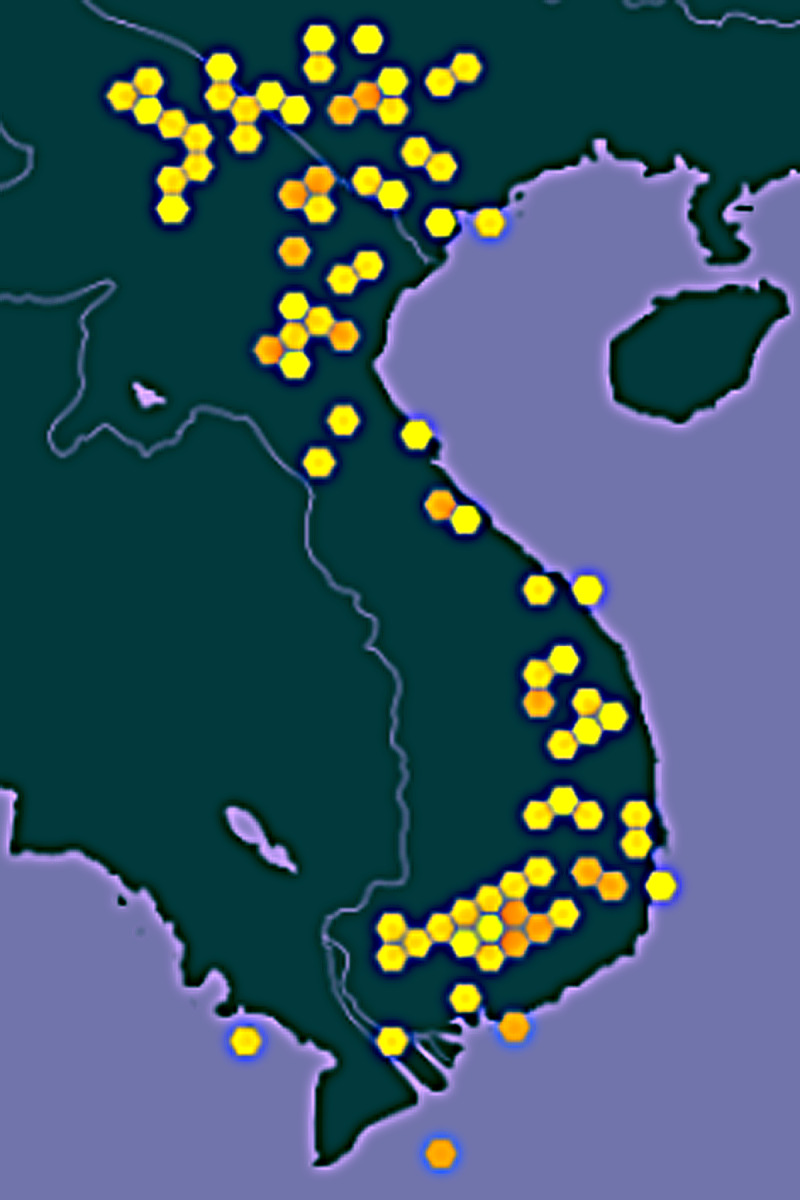
Geographical distribution of occurrence records.

The geographic distribution of samples lies within the geographical area specified by the coordinates 8.6877-23.20476N and 102.2375-109.29083E. It contains 164 individual geographical localities, determined usually with an accuracy of 10 to 1000 m (with a few samples to 5000 m). For each locality, from 1 to 12 species of small mammals were recorded. The study area covers the whole of Vietnam and, currently, the dataset also includes a few samples from central Laos (Fig. 4).

## Sampling methods

### Study extent

Small terrestrial mammals occurrence records have been collected from various sources: field data gathered by the authorover 15 years, including ~ 1500 capture records and ~ 900 other samples (records) presented to the author by colleagues and also obtained during a number of expeditions; the dataset also included some records from samples from collections of the Department of Theriology of Zoological Museum of Moscow State University (ZMMU, Moscow). In general, samples were collected from 164 geographically attributed sites from 35 provinces and territories of Vietnam and Khammouane Province of Laos.

### Sampling description

The presented materials are based on genetic collections (total DNA samples) under the author's supervision. The data combine 2408 records including 1688 Muridae, 174 Sciuridae, 10 Platacanthomyidae, 252 Spalacidae, 171 Tupaiidae along with 113 genus-specified sample records (Table 1). This collection also includes a number of insectivorous and small carnivora samples, which currently arenot included in the dataset presented here. Although the records correspond and partially overlap with the ZMMU museum samples (skulls, skins, ethanol preserved bodies), as well as othersstored at the Zoological Institute of Russian Academy of Sciences (ZIN RAS, Saint-Petersburg), they do not cover completely the collections of these museums, but contain data only about the samples (treated here as records) deposited by the author or with his participation.

### Quality control

Only the samples for which the location could be determined closely were included in the dataset. A significant part of the records was taken from our publications where corresponding data first appeared. For others not yet published, field-note records and supporting samples information have been used. For a few samples brought into studies from third party institutions (Museums and various persons), we performed geosearch by Yandex.geo and Google Maps to find the nearest geographic point found in supporting information to obtain the geoposition coordinates with a resolution not greater than 1000 m.

**baseOfRecord**: Any samples and data records with an unknown baseOfRecord were removed from our dataset to ensure that all accepted records were based on confirmed observation data (findings and sampling).

**ScientificName**: We followed the most modern taxonomy conception as realised by Wilson and Reeder's "Mammal Species of The World" 3rd edition (Wilson and Reeder 2005) and available under the supervision of the Smithsonian Institution (Washington, USA) from http://www.departments.bucknell.edu/biology/resources/msw3/ with most recent novelties and corrections made as registered in ZooBank (The Official Registry of Zoological Nomenclature https://zoobank.org). Although the author has reasons to believe that some species and groups still need taxonomic revision, which will require a corresponding revision of applied nomenclature, the specific names are given in accordance with the actual taxonomic acts as of 15 September 2022.

**eventDate**: Data within columns were edited using controlled vocabulary and the most modern Darwin Core standards (Wieczorek et al. 2012). The data value was usually presented as day of capture if available, although, for a few museums samples, it was given roughly as month or as year.

## Geographic coverage

### Description

The most significant part of the occurrence records 99.25% (2391) are located in Vietnam, a few samples also coming from Laos: 0.25% (18) records, the most northern point being recorded in Vietnam, Ha Giang Province, Bach Dich District, Muong Village, 23.20476 N, 105.0371 E), western (Dien Bien Province, Muong Nhe District, 22.3650 N, 102.2375 E) and eastern (Tay Ninh Province, Tan Binh, 13.1500 N, 109.9500 E). The southernmost occurrence records were located in Con Son Island, Ba Ria-Vung Tau Province, Con Dao District (8.6877 N, 106.5888 E).

### Coordinates

8.6877 N, 106.5888 E and 23.20476 N, 105.0371 E Latitude; 22.3650 N, 102.2375 E and 13.1500 N, 109.9500 E Longitude.

## Taxonomic coverage

### Description

The presented data cover almost completely the fauna of Muridae, Platacanthomyidae and Spalacidae of the region, as well as the largest genera of Sciuridae and includes distribution ranges of 58 species of rodents (41 out of Muridae, 15 out of Sciuridae, three out of Spalacidae and one from Platacanthomyidae) and two species of Scandentia (Table 1).

### Taxa included

**Table:** 

Rank	Scientific Name	Common Name
kingdom	Animalia	Animals
subkingdom	Eumetazoa	
phylum	Chordata	
subphylum	Vertebrata	
class	Mammalia	mammals
subclass	Theria	true mammals
order	Rodentia	rodents
superfamily	Muroides	muroids
family	Muridae	mice and rats
family	Sciuridae	squirrels
subfamily	Sciurinae	true squirrels
subfamily	Pteromiinae	flying squirrels
family	Platacanthomyidae	oriental dormice
family	Spalacidae	zokors
subfamily	Rhizomiinae	bamboo rats
order	Scandentia	tree shrews
family	Tupaiidae	tree shrews

## Temporal coverage

**Data range:** 1987-1-01 – 2022-5-26.

## Collection data

### Collection name

IPEE RAS Vietnamese rodents’ collection

### Collection identifier

IPEE RAS Rodents

### Parent collection identifier

IPEE RAS

### Specimen preservation method

Alcohol total DNA preservation.

### Curatorial unit

Laboratory of Tropical Ecology, Alexander E. Balakirev PhD.

## Usage licence

### Usage licence

Other

### IP rights notes

aCC BY 4.0. Samples and additional information available upon request. See individual records for usage rights.

## Data resources

### Data package title

IPEE RAS Vietnamese rodents’ collection

### Number of data sets

1

### Data set 1.

#### Data set name

Vietnamese rodents’ collection

#### Data format

Darwin Core Archive

#### Download URL

https://www.gbif.org/dataset/dc8da9fe-ce38-44b1-9242-2f8f31f5bb19

#### Data format version

1.2

#### Description

The data combine 2408 records including 1688 Muridae, 174 Sciuridae, 10 Platacanthomyidae, 252 Spalacidae and 171 Tupaiidae along with 113 genus-specified sample records (Balakirev 2022). For the samples, the author provided taxonomic information and information about date and sampling locality, age and sex of animals, along with information about the persons who sampled and investigated them and corresponding data about samples like field numbers, museum number, type status and GenBank IDs, if available. Official institutions where collection were available and contact information are also provided.

**Data set 1.** 

Column label	Column description
recordedBy	The person who initially made records about the sample (animal, record), usually original collector.
occurrenceID	In this dataset, occurrence records use the ID number from its holding facility when applicable. Occurrence records that did not have a unique ID were given their own unique observation ID.
basisOfRecord	The specific nature of the data record. We used a Darwin Core controlled vocabulary for our basisOfRecord that included "GeneticSample".
eventDate	The date-time or interval during which an Event occurred. For occurrences, this is the data-time when the event was recorded (animal was trapped).
identifiedBy	A list of names of peoples who assigned the Taxon to the subject.
identificationVerificationStatus	A categorical indicator of the extent to which the taxonomic identification has been verified to be correct.
scientificName	The full scientific name, with authorship and date information, if known.
identificationRemarks	Comments or notes about the taxon or name.
sex	The sex of the biological individual represented in the Occurrence.
lifeStage	The age class or life stage of the Organism at the time the Occurrence was recorded.
taxonRank	The taxonomic rank of the most specific name in the scientificName.
kingdom	The full scientific name of the kingdom in which the taxon is classified.
phylum	The full scientific name of the phylum in which the taxon is classified.
class	The full scientific name of the class in which the taxon is classified.
order	The full scientific name of the order in which the taxon is classified.
family	The full scientific name of the family in which the taxon is classified.
genus	The full scientific name of the genus in which the taxon is classified.
countryCode	The standard code for the country in which the Location occurs. VN for Vietnam and LA for Laos.
stateProvince	The name of the next smaller administrative region than country (state, province, canton, department, region etc.) in which the Location occurs.
county	The full, unabbreviated name of the next smaller administrative region than stateProvince (county, shire, department etc.) in which the Location occurs.
locality	The specific description of the place. This term may contain information modified from the original to correct perceived errors or to standardise the description.
verbatimLocality	The original textual description of the place.
habitat	A category or description of the habitat in which the Event occurred (occurrence has been placed, usually type of enveronment).
fieldNumber	An identifier given to the event (animal) in the field.
decimalLatitude	The latitude of the location from which the catalogued item was collected, expressed in decimal degrees.
decimalLongitude	The longitude of the location from which the catalogued item was collected, expressed in decimal degrees.
verbatimElevation	The original description of the elevation (altitude above sea level, in metres) of the Location.
geodeticDatum	The ellipsoid, geodetic datum or spatial reference system (SRS) upon which the geographic coordinates given in decimalLatitude and decimalLongitude are based.
georeferenceSources	A list (concatenated and separated) of maps, gazetteers or other resources used to georeference the Location, described specifically enough to allow anyone to use the same resources.
coordinateUncertaintyInMetres	The horizontal distance (in metres) from the given decimalLatitude and decimalLongitude describing the smallest circle containing the whole of the Location.
bibliographicCitation	A bibliographic reference for the resource as a statement indicating how this record should be cited (attributed) when used.
typeStatus	A list (concatenated and separated) of nomenclatural types (type status, typified scientific name, publication) applied to the subject.
institutionCode	The name (or acronym) in use by the institution having custody of the object(s) or information referred to in the record.
rightsHolder	Contact person and organisation holding rights for data published and their use for any purposes.
associatedSequences	GenBank IDs associated with records (if available).
minimumElevationInMetres	Minimum level of the elevation (altitude, above sea level, in metres) of the Location.
maximumElevationInMetres	Miximum level of the elevation (altitude, above sea level, in metres) of the Location.
catalogNumber	Museums' catalogue number associated with records, if available (ZIN is Zoological Institute of Russian Academy of Sciences, Saint-Petersburg, Russia; ZMMU is Zoological Museum of Moscow State Universoty, Moscow, Russia).

## Figures and Tables

**Figure 1.**
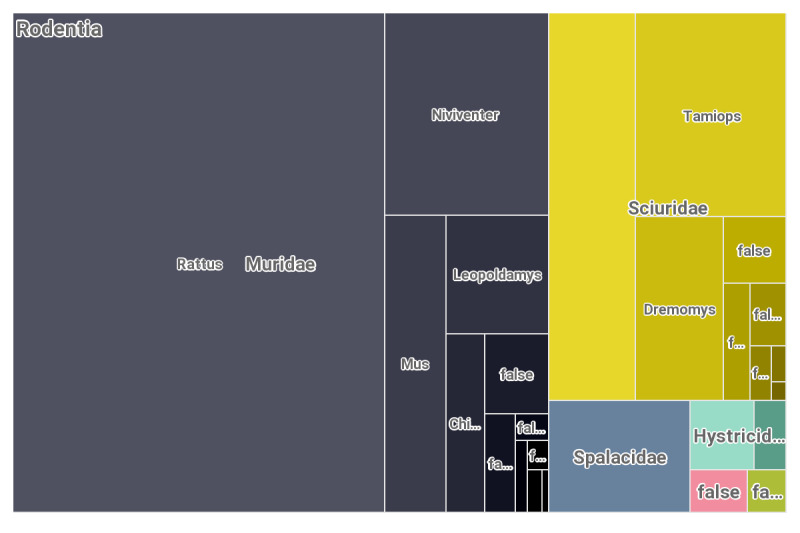
The rodents' records registered for Vietnam in the GBIF database up to the date of this publication.

**Figure 2.**
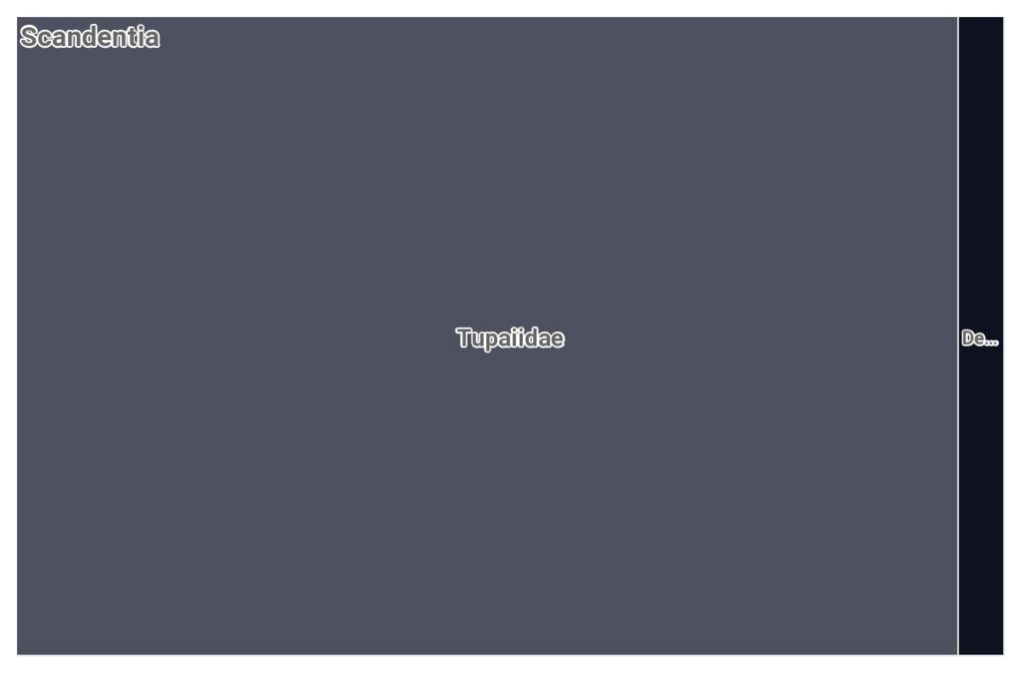
The Scandentia records registered for Vietnam in GBIF database up to the date of this publication.

**Figure 3.**
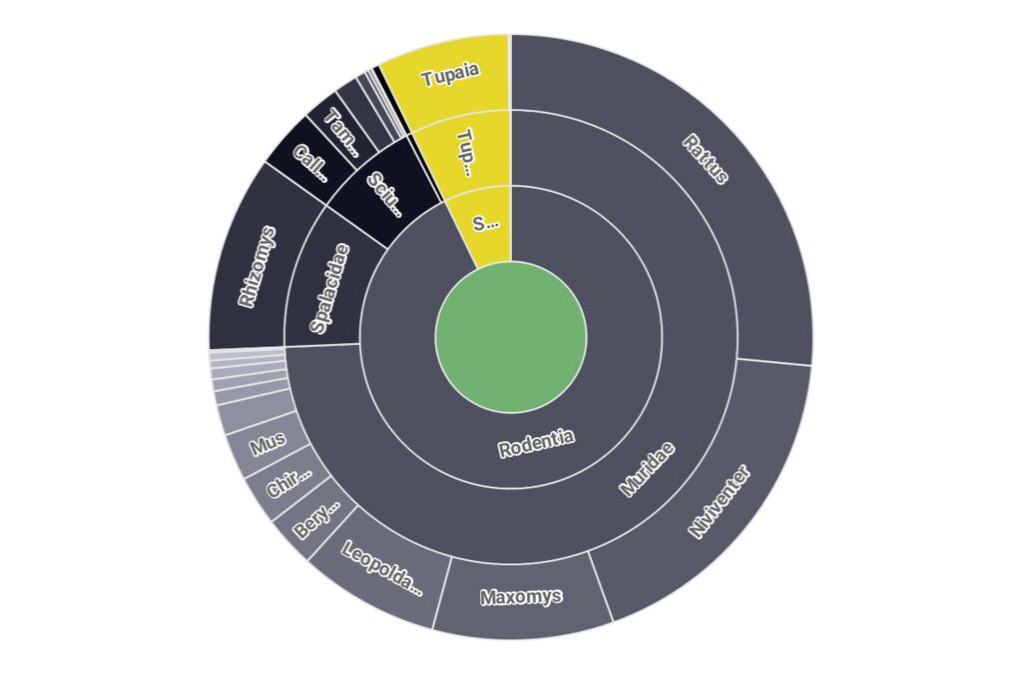
New records taxonomical distribution.

**Table 1.** Database content by species.

	**Species**	**Number of records**
Muridae	*Bandicotaindica* (Bechstein, 1800)	19
	*Bandicotasavilei* (Thomas, 1916)	20
	*Berylmysberdmorei* (Blyth, 1851)	31
	*Berylmysbowersi* (Anderson, 1879)	36
	*Chiromyscuschiropus* (Thomas, 1891)	22
	*Chiromyscuslangbianis* (Robinson and Kloss, 1922)	31
	*Chiromyscusthomasi* Balakirev, Abramov and Rozhnov, 2014	16
	*Chiropodomysgliroides* (Blyth, 1856)	18
	*Dacnomysmillardi* Thomas, 1916	10
	*Hapalomysdelacouri* Thomas, 1927	1
	*Hapalomyssuntsovi* Abramov, Balakirev and Rozhnov, 2017	14
	*Leopoldamysedwardsi* (Thomas, 1882)	46
	*Leopoldamysherberti* (Kloss, 1916)	91
	*Leopoldamysmilleti* (Robinson and Kloss, 1922)	4
	*Leopoldamysneilli* (J.T.Marshall Jr., 1976)	37
	*Maxomysmoi* (Robinson and Kloss, 1922)	30
	*Maxomyssurifer* (Miller, 1900)	202
	*Muscaroli* Bonhote, 1902	3
	*Muscervicolor* Hodgson, 1845	5
	*Muscookii* Ryley, 1914	2
	*Musmusculus* Linnaeus, 1758	11
	*Muspahari* Thomas, 1916	15
	*Niviventerbukit* (Bonhote, 1903)	67
	*Niviventerconfucianus* (Milne-Edwards, 1871)	4
	*Niviventerfulvescens* (Gray, 1847)	25
	*Niviventerlotipes* (Allen, 1926)	32
	*Niviventermekongis* (Robinson and Kloss, 1922)	240
	*Niviventerniviventer* (Hodgson, 1836)	15
	*Niviventertenaster* (Thomas, 1916)	27
	*Rattusandamanensis* (Blyth, 1860)	232
	*Rattusargentiventer* (Robinson and Kloss, 1916)	20
	*Rattusexulans* (Peale, 1848)	48
	*Rattuslosea* (Swinhoe, 1871)	10
	Rattus nitidus (Hodgson, 1845)	16
	*Rattusnorvegicus* Berkenhout, 1769	33
	*Rattusosgoodi* Musser and Newcomb, 1985	13
	*Rattussp* (type IV)	69
	*Rattustanezumi* (Temminck, 1844)	145
	*Saxatilomyspaulinae* Musser et al., 2005	10
	*Tonkinomysdaovantieni* Musser, Lunde and Nguyen Truong Son, 2006	14
	*Vandeleuriaoleracea* (Bennett, 1832)	4
Sciuridae	*Belomyspearsonii* (Gray, 1842)	1
	*Callosciuruserythraeus* (Pallas, 1779)	28
	*Callosciurusfinlaysonii* (Horsfield, 1823)	24
	*Callosciurusinornatus* (Gray, 1867)	26
	*Dremomysornatus* Thomas, 1914	13
	*Dremomysrufigenis* (Blanford, 1878)	18
	*Hylopetesphayrei* (Blyth, 1859)	2
	*Hylopetesspadiceus* (Blyth, 1847)	1
	*Menetesberdmorei* (Blyth, 1849)	13
	*Petauristaalborufus* (Milne-Edwards, 1870)	2
	*Petauristaelegans* (Müller, 1840)	1
	*Petauristaphilippensis* (Elliot, 1839)	1
	*Tamiopsmaritimus* (Bonhote, 1900)	36
	*Tamiopsrodolphii* (Milne-Edwards, 1867)	2
	*Tamiopsswinhoei* (Milne-Edwards, 1874)	6
Platacanthomyidae	*Typhlomyschapensis* Osgood, 1932	10
Spalacidae	*Rhizomyspruinosus* Blyth, 1851	236
	*Rhizomyssinensis* Gray, 1831	6
	*Rhizomyssumatrensis* (Raffles, 1821)	10
Order Scandentia	*Dendrogalemurina* (Schlegel and Müller, 1843)	2
Tupaiidae	*Tupaiabelangeri* (Wagner, 1841)	169
	Genus specified samples	113
**Overall**		**2408**
